# Genome-wide methylation profiling of ovarian cancer patient-derived xenografts treated with the demethylating agent decitabine identifies novel epigenetically regulated genes and pathways

**DOI:** 10.1186/s13073-016-0361-5

**Published:** 2016-10-20

**Authors:** Tushar Tomar, Steven de Jong, Nicolette G. Alkema, Rieks L. Hoekman, Gert Jan Meersma, Harry G. Klip, Ate GJ van der Zee, G. Bea A. Wisman

**Affiliations:** 1 Department of Gynecologic Oncology, University of Groningen, University Medical Center Groningen, PO Box 30001, Groningen, 9700 RB The Netherlands; 2 Medical Oncology, Cancer Research Center Groningen, University of Groningen, University Medical Center Groningen, Groningen, The Netherlands

## Abstract

**Background:**

In high-grade serous ovarian cancer (HGSOC), intrinsic and/or acquired resistance against platinum-containing chemotherapy is a major obstacle for successful treatment. A low frequency of somatic mutations but frequent epigenetic alterations, including DNA methylation in HGSOC tumors, presents the cancer epigenome as a relevant target for innovative therapy. Patient-derived xenografts (PDXs) supposedly are good preclinical models for identifying novel drug targets. However, the representativeness of global methylation status of HGSOC PDXs compared to their original tumors has not been evaluated so far. Aims of this study were to explore how representative HGSOC PDXs are of their corresponding patient tumor methylome and to evaluate the effect of epigenetic therapy and cisplatin on putative epigenetically regulated genes and their related pathways in PDXs.

**Methods:**

Genome-wide analysis of the DNA methylome of HGSOC patients with their corresponding PDXs, from different generations, was performed using Infinium 450 K methylation arrays. Furthermore, we analyzed global methylome changes after treatment of HGSOC PDXs with the FDA approved demethylating agent decitabine and cisplatin. Findings were validated by bisulfite pyrosequencing with subsequent pathway analysis. Publicly available datasets comprising HGSOC patients were used to analyze the prognostic value of the identified genes.

**Results:**

Only 0.6–1.0 % of all analyzed CpGs (388,696 CpGs) changed significantly (*p* < 0.01) during propagation, showing that HGSOC PDXs were epigenetically stable. Treatment of F3 PDXs with decitabine caused a significant reduction in methylation in 10.6 % of CpG sites in comparison to untreated PDXs (*p* < 0.01, false discovery rate <10 %). Cisplatin treatment had a marginal effect on the PDX methylome. Pathway analysis of decitabine-treated PDX tumors revealed several putative epigenetically regulated pathways (e.g., the Src family kinase pathway). In particular, the C-terminal Src kinase (*CSK*) gene was successfully validated for epigenetic regulation in different PDX models and ovarian cancer cell lines. Low *CSK* methylation and high *CSK* expression were both significantly associated (*p* < 0.05) with improved progression-free survival and overall survival in HGSOC patients.

**Conclusions:**

HGSOC PDXs resemble the global epigenome of patients over many generations and can be modulated by epigenetic drugs. Novel epigenetically regulated genes such as *CSK* and related pathways were identified in HGSOC. Our observations encourage future application of PDXs for cancer epigenome studies.

**Electronic supplementary material:**

The online version of this article (doi:10.1186/s13073-016-0361-5) contains supplementary material, which is available to authorized users.

## Background

Ovarian cancer is the fifth most common cancer type in women and is the most lethal gynecologic malignancy [1]. The most abundant histological subtype of ovarian cancer, high-grade serous ovarian cancer (HGSOC), is characterized by mutations in a few genes, mainly *TP53* and *BRCA1/2* [2]. Therefore, changes in the epigenome, like DNA methylation and histone modifications, may play an important role in the biological behavior of the disease. Aberrant DNA methylation patterns are universally observed in HGSOC and are known to frequently affect gene regulation involved in cancer-related processes [2–5]. Since epigenetic modifications, including DNA methylation, are reversible in nature, these epigenetic alterations have emerged as attractive targets for epigenetic therapy for cancer [6, 7].

Effective treatment of cancer relies on the identification of key molecular targets of cancer growth and subsequent development of therapeutic agents against these targets. This in turn mainly depends on preclinical research and predictive model systems. Recent genomic analyses have shown that most commonly used HGSOC cell lines, like SKOV3 and A2780, are less representative models of HGSOC [8, 9]. Recently, patient-derived xenografts (PDXs), i.e., patient tumor tissues transplanted directly into immune-deficient mice, have appeared as better representative preclinical models [10]. They recapitulate the histological type and maintain the genomic features and reminiscent heterogeneity of corresponding patients’ primary tumors [11–13]. Furthermore, results from treatment of ovarian cancer PDXs have a good predictive value for standard platinum-based chemotherapy and novel therapeutic agents [14–16]. Although several comparative gene expression and mutational studies have been performed for HGSOC PDXs, comparable studies on the epigenome are not available. Until now, only a few small studies in other tumor types have compared genome-wide DNA methylation of PDXs with their corresponding solid patient tumors [17–19].

In the present study, we first compared genome-wide DNA methylation patterns in different generations of HGSOC PDX tumors and their corresponding primary tumors using Infinium 450 K methylation arrays. Furthermore, we analyzed global methylome changes after treatment of HGSOC PDXs with decitabine (DAC), a DNA demethylating agent, and cisplatin, as platinum-containing chemotherapy is standard of care in first-line treatment of HGSOC. The findings were validated and pathway analysis was performed.

## Methods

### PDX establishment and treatment

PDXs were established as described previously [12]. Briefly, after patients gave informed consent, HGSOC specimens were obtained at primary debulking surgery (patient 36 and -37) or at interval surgery (patient- 56). The clinicopathological features of each patient are provided in Additional file 1: Figure S1a. Tumor fragments were cut into pieces of 3 × 3 × 3 mm^3^ and implanted in 6–12-week-old female NOD.Cg-Prkdcscid Il2rgtm1Wjl/SzJ mice (NSG mice, internal breed, Central Animal Facility, University Medical Center Groningen). Periodic two-dimensional tumor measurement was carried out using a slide vernier caliper and when the tumor size reached >1 cm^3^, tumors were harvested and were either directly propagated into a further generation or snap frozen in liquid nitrogen for storage along with a piece for formalin fixation. To investigate global DNA methylation changes related to establishment of PDX models from primary HGSOC, we implanted primary tumors of three different HGSOC patients (patients 36, 37, and 56) into the flanks of NSG mice (PDX-36, -37 and -56) and tumors were propagated for up to three generations (F1, F2, and F3) (Additional file 1: Figure S1b). The histology of primary tumors and PDX tumors was analyzed by an experienced gynecologic pathologist.

Mice with F3 PDX tumors were used for treatment. When tumor size reached up to 200 mm^3^ in size, they were treated with either saline vehicle (n = 3), demethylating agent DAC (n = 3, 2.5 mg/kg three times/week), or cisplatin (n = 3, 4 mg/kg/week) for up to 4 weeks (Additional file 2: Figure S2a). During treatment, mice were regularly checked for welfare and tumor growth (three times a week). After completion of treatment, tumors were harvested and excised into two pieces, one of which was fixed in formalin and the other snap frozen in liquid nitrogen.

### Cell line culturing

The ovarian cancer cell lines CaOV3, SKOV3, OVCAR3, PEA1, PEA2, PEO14, PEO23, A2780, C30, Cp70, and IGROV1 were used for in vitro validation. The media used and culture conditions of cell lines are described in Additional file 3: Table S1. All cells were grown at 37 °C in a humidified atmosphere with 5 % CO_2_ and were detached with 0.05 % trypsin in phosphate-buffered saline (PBS; 0.14 mM NaCl, 2.7 mM KCl, 6.4 mM Na_2_HPO_4_, 1.5 mM KH_2_PO_4_, pH = 7.4). The authenticity of all cell lines was verified by DNA short tandem repeat analysis (Baseclear, Leiden, The Netherlands). Cells at 40–50 % confluency were treated with DAC (1 μM) for 72 h and the medium was replenished with DAC every day. For cisplatin and carboplatin, cells were treated for 72 h without any daily media replenishment. After 72 h, cells were trypsinized and processed for RNA and DNA isolation.

### DNA extraction and bisulfite modification

For DNA isolation, representative frozen blocks of each sample or cells were retrieved. Frozen sections of 10 μm were cut with periodic 4 μm sections for hematoxylin and eosin staining to evaluate the vital tumor cell percentage. DNA of all samples was isolated using standard salt-chloroform extraction and isopropanol precipitation. Precipitated DNA was resuspended in Tris-EDTA buffer (10 mM Tris, 1 mM EDTA, pH = 8.0). Genomic DNA was amplified in a multiplex PCR according to the BIOMED-2 protocol to check the structural integrity of the DNA. DNA concentrations at A_260_ were measured using the Nanodrop ND-1000 Spectrophotometer (Thermo Scientific, Waltham, MA, USA). A_260/280_ ratio of >1.8 was required for all samples. Subsequently, bisulfite conversion of all samples was done as described before [20] using an EZ DNA methylation™ kit (Zymo Research, Orange, CA, USA) as per the manufacturer’s protocol using 1 μg of DNA.

### Genome-wide methylation Infinium 450 K array

To analyze the methylation status, the Infinium HumanMethylation450K (HM450K) platform consisting of 485,512 CpG sites was used. The assay was carried out as described [21]. In brief, 4 μl of bisulfite-converted DNA (~150 ng) was used in the whole-genome amplification reaction. After amplification, DNA was fragmented enzymatically, precipitated, and re-suspended in hybridization buffer. All subsequent steps were performed following the standard Infinium protocol (User Guide part #15019519 A). Fragmented DNA was dispensed onto the HumanMethylation450 BeadChips and hybridization was performed in a hybridization oven for 20 h. After hybridization, the array was processed through a primer extension and an immunohistochemistry staining protocol to allow detection of a single-base extension reaction. Finally, BeadChips were coated and then imaged on an Illumina iScan. Methylation levels were computed from raw iDAT files using R (http://www.R-project.org) with different R packages, including MinFi [22] and ChAMP [23].

### HM450K data processing

Raw iDAT files were imported using the Bioconductor (http://www.bioconductor.org) suite for R. Methylation levels, β, were represented according to the following equation:

$$ \upbeta = \mathrm{M}/\left(\mathrm{M} + \mathrm{U} + 100\right) $$

where M represents the signal intensity of the methylated probe and U represents the signal intensity of the unmethylated probe. Illumina recommends adding the constant 100 to the denominator to regularize β values with very low values for both M and U. Probe dye bias was normalized using built-in control probes. Probes with a detection *p* value <0.01 were omitted. Finally, probes from X and Y chromosomes, single nucleotide polymorphism probes and possible cross-hybridized probes were excluded, leaving 468,665 unique probes. Furthermore, host mouse DNA can potentially contaminate the signal from human PDX tumor if stromal and endothelial cells of murine origin are extracted with the tumor. To eliminate these confounders in our methylation analysis, an additional mouse tail sample was processed on the 450 K array and 47,240 probes were removed from the downstream analysis after passing a detection *p* value threshold of 0.01. After probe filtering and removal of mouse specific probes, normalization was performed using a beta-mixture quantile (BMIQ) normalization method for correcting Infinium I/II probe type bias in Illumina Infinium 450 K data [24]. Furthermore, we analyzed and corrected for batch effects using the *ComBat* function of the SVA package [25]. Subsequently, we compared the β values of the remaining 392,317 autosomal CpG probes for further analysis (Additional files 1 and 2: Figures S1c and S2b).

Besides PDX tumors, we also used SKOV3 cells treated with a high dose of DAC (1 μM) for 72 h as described in the “Cell line culturing” section. Since SKOV3 is one of the most DAC-sensitive ovarian cancer cell lines, we used it as a positive control for DAC-induced demethylation effects. The ultimate goal was to use these data as a filter to screen the DAC-mediated demethylation-sensitive genes for further in vitro validation. Results of genome-wide methylation of SKOV3 were also processed in a similar way as for PDX tumors. For annotation of probe region, we used UCSC-based annotations in the context of genomic compartment and CpG islands. Further, an additional biologically relevant probe annotation was applied based on CpG enrichment, known as “HIL” CpG classes, consisting of high-density CpG island (HC), intermediate-density CpG island (IC), and non-island (LC).

### Bisulfite pyrosequencing

Bisulfite pyrosequencing was performed as described previously [26]. Briefly, bisulfite-treated DNA was amplified using a PyroMark PCR kit (Qiagen, Hilden, Germany). PCR and cycling conditions were according to the kit manual. All pyrosequencing primers (PCR primers and sequencing primers) were based on the selected candidate 450 K array CpG probe using PyroMark Assay Design software (Qiagen). The amplification protocol was performed according to Collela et al. [27] using a universal primer approach. The biotinylated PCR products were captured using 1.0 μl streptavidin-coated sepharose high-performance beads (GE Healthcare, Little Chalfont, UK). The immobilized products were washed with 70 % alcohol, denatured with PyroMark denaturation solution (Qiagen), and then washed with PyroMark wash buffer (Qiagen). The purified PCR product was then added to 25 μl PyroMark annealing buffer (Qiagen) containing 0.3 μM sequencing primers for specific genes (all primers and their sequences are available on request). Finally, pyrosequencing was performed using the Pyromark Q24 MD system (Qiagen) according to the manufacturer’s instructions using the PyroGold Q24™ Reagent kit (Qiagen). Data were analyzed and quantified with the PyroMark Q24 software version 2.0.6 (Qiagen).

### Total RNA isolation, cDNA synthesis and quantitative RT-PCR

Quantitative reverse transcriptase (qRT)-PCR was performed as described previously [28]. Total RNA was isolated from frozen tissue blocks and cell lines similarly to as described for DNA extraction. RNA was isolated using a RNeasy mini kit (Qiagen) according to the instructions of the manufacturer. RNA was analyzed quantitatively using a Nanodrop and integrity was checked using electrophoresis on agarose gel. Total RNA (1 μg) was used for cDNA synthesis by RNase H+ reverse transcriptase using an iScript cDNA synthesis kit (BioRad, Hercules, CA, USA) as per the manufacturer’s instructions. qRT-PCR was performed in an ABI PRISM 7900HT Sequence Detector (Applied Biosystems, Foster City, CA, USA) with the iTaq SYBR Green Supermix with Rox dye (Biorad, Hercules, CA, USA). Amplification was performed with the following cycling conditions: 5 min at 95 °C, and 40 two-step cycles of 15 s at 95 °C and 25 s at 60 °C. The reactions were analyzed by SDS software (version 2.4, Applied Biosystems). The threshold cycles (Ct) were calculated and relative gene expression was analyzed after normalizing for GAPDH, a house-keeping gene. qRT-PCR primer sequences are available on request.

### Statistical analysis

After performing probe filtering, normalization and batch effect correction, we identified the differentially methylated CpG sites using Linear Models for Microarray Data (LIMMA) analysis [29]. Since for beta-distributed data like DNA methylation β values the variance is associated with the mean (heteroscedasticity) [30], we cannot apply linear model-based methods without transforming the data properly (logit transformed). Therefore, normalized 450 K probe β values were converted to M values using the beta2m function [30]. The unpaired statistical analysis was performed using the eBayes function of the Limma package [31]. The average DNA methylation of bisulfite pyrosequencing and RNA expression levels were presented as mean ± standard deviation (SD) using the GraphPad Prism version 6.04 (GraphPad for Science, San Diego, CA, USA). Statistical significance was calculated by two-way Student’s *t*-test and multiple comparisons between different groups were performed by one-way ANOVA with Bonferroni post-test, unless otherwise mentioned in the respective figure legends. For selection of differentially methylated CpG sites the cutoff was *p* < 0.01, while other analyses are described in the respective figure legends with appropriate symbolic representation. As a positive control for DAC-induced genome-wide demethylation, SKOV3 cells showed a higher percentage (39.3 %) of CpG sites being demethylated (Additional file 2: Figure S2b, e) in comparison with DAC-treated PDX-36. These DAC-sensitive CpG sites from SKOV3 cells were also used for identification of epigenetically regulated genes and pathways for in vitro validation.

### Cluster analysis

Principal component analysis was performed on BMIQ normalized data. Pre-processed, filtered, and normalized autosomal CpG probes were used for unsupervised clustering of Illumina 450 K data. Different clustering algorithms and number of clusters were investigated extensively, including k-means and hierarchical clustering approaches using average linkage methodology. Further, supervised clustering analysis was performed on significant probes after LIMMA analysis on treatment groups using hierarchical clustering with the average linkage method.

### Gene ontology analysis

Functional gene ontology (GO) term enrichment analysis was performed with the DAVID tool [32] using DAC-sensitive genes (n = 822) on *Homo sapiens* as species background. We restricted the analysis to the biological process category and selected GO terms with enrichment (*p* ≤ 0.01). Data visualization was carried out using REVIGO (http://revigo.irb.hr/index.jsp) [33].

### Web-based tools for networks and pathway analysis

WebGestalt (WEB-based GEne SeT AnaLysis Toolkit) [34] was used as the web-based tool for prediction of associated pathway and gene function using the list of DAC-sensitive genes in PDX tumors (n = 822). Parameters used for analysis were: organism, *H. sapiens*; ID type, gene_symbol; reference set, Entrez gene; significance level, 0.001; statistics test, hypergeometric; multiple testing corrections, Bonferroni Hedgehog test; minimum number of genes for enrichment, 3. Pathway analyses were performed using KEGG, Wiki pathways, and pathways from common databases. Genes related to pathways found in at least two of the databases were included for the final networks using the Gene Multiple Association Network Integration Algorithm (GeneMANIA; http://www.genemania.org/). This analysis builds a gene integration network incorporating physical and predicted interactions, co-localization, shared pathways, and shared protein domains.

### Prognostic evaluation of *CSK* methylation and expression on clinical data

Methylation data of the AOCS study group (http://www.aocstudy.org) was downloaded from the NCBI GEO portal using GEO accession GSE65820 (http://www.ncbi.nlm.nih.gov/geo/query/acc.cgi?acc =GSE65820) as mentioned in Patch et al. [35]. The clinical data of patients was downloaded from the ICGC data portal (https://dcc.icgc.org/). Data were normalized using a BMIQ normalization as described previously [24]. The *CSK* methylation probe (cg00516515) identified in the PDX methylation analysis was used for further analysis. The methylation cutoff between low and high methylation was set at 0.9 based on the median β value (0.90, range 0.78–0.96). This resulted in 89 patients (31 high and 58 low methylation) for progression-free survival (PFS) analysis (a proxy for sensitivity to platinum-containing chemotherapy) and 91 patients (32 high and 59 low methylation) for overall survival (OS) analysis using the Cox proportional hazard model.

Prognostic validation of *CSK* expression level was performed on publicly available datasets obtained from an online tool [36] for genome-wide validation that can be accessed at http://kmplot.com/ovar. This online portal only contains data from publications that comprise normalized microarray gene expression data, clinical survival information, and at least 20 patients. For our prognostic analysis, data were derived from analysis using KM plotter [36] in October 2015, in which we selected only advanced stage (III and IV) HGSOC cancer patients who received platinum therapy. This resulted in 633 patients for PFS analysis and 656 patients for OS analysis using a Cox proportional hazard model with *CSK* probe (probe ID 202329_at). With an expression range of *CSK* probe (74–2566), the auto cutoff value of 567 for PFS analysis and 580 for OS analysis was used, based on the computation of upper and lower quartiles with default settings of the portal [36].

## Results

### Genome-wide DNA methylation comparison of HGSOC primary and PDX tumors

**Fig. 1:**
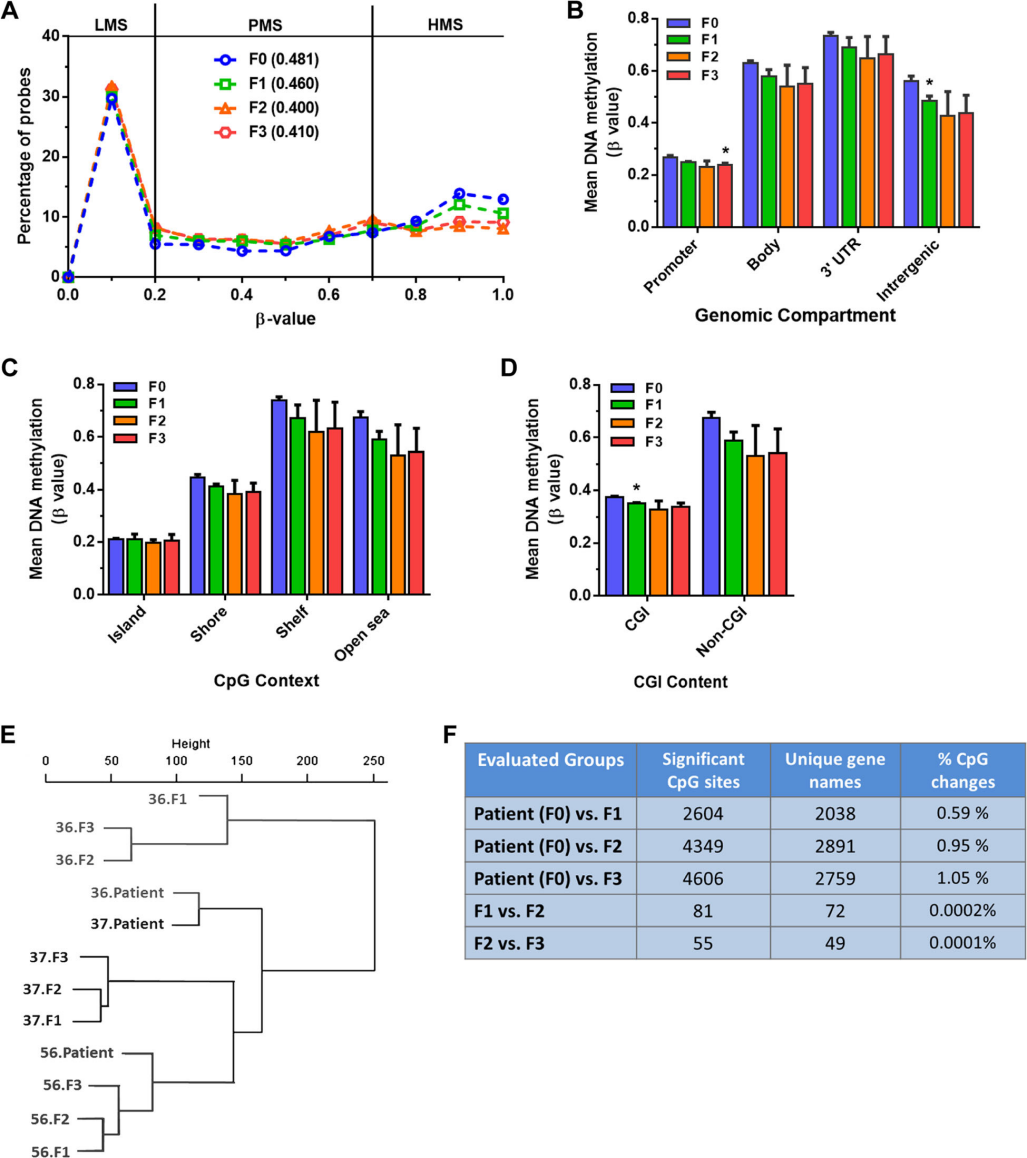
Distribution of methylated CpG sites in HGSOC primary tumors and three generations of their corresponding PDX tumors. **a** β values are grouped in 0.1 increments and the percentage of probes is represented for each sample type (from patients (*F0*) to third generation PDX tumors (*F3*)). The mean β value for each sample type is shown between *parentheses*. Lowly, partially and highly methylated sites are indicated as *LMS*, *PMS*, and *HMS*, respectively. **b**–**d** DNA methylation level of each sample type according to the genomic compartment (**b**), CpG context (**c**) and CpG island (*CGI*) content (**d**). Each *bar* represents mean DNA methylation β value ± SD; *p < 0.01. **e** Unsupervised clustering dendrogram showing the relationship of CpG probes between all the sample types. **f** The number of significant CpG sites in comparison with different sample types and their percentage compared to total CpG sites analyzed

Genome-wide DNA methylation of HGSOC primary tumors (F0) and different PDX generations (F1, F2, and F3) from three patient-derived PDX models (PDX-36, -37, and -56) was studied. We analyzed up to generation F3 because this PDX generation is regarded as being stable and can be used for testing therapeutic agents [10, 12, 37]. Marginal differences were found in mean genome-wide DNA methylation (β value) from primary tumors (F0 = 0.481) to PDX.F3 tumors (F3 = 0.410). This difference can be largely explained by the more abundant presence of “highly methylated sites” (HMS; β values >0.7) and less “partially methylated sites” (PMS; β values 0.2–0.7) in primary tumors (F0) compared to PDX tumors (F1, F2, and F3) (Fig. 1a; Additional file 1: Figure S1d). Further, we comparatively analyzed all DNA methylation probes based on genomic compartment (Fig. 1b), CpG context (Fig. 1c), CpG island content (Fig. 1d), and HIL CpG classes (high-density CpG island (HC), intermediate-density CpG island (IC), and non-island (LC)) based on CpG enrichment [38] (Additional file 1: Figure S1e). Notably, no major methylation changes were found for the mean methylation β value of the probes at different regions of CpG islands among all samples. The largest differences in methylation levels were found between promoter regions of F0 primary tumors and F3 PDX tumors and between intragenic regions of F0 primary tumors and F1 PDX tumors (Fig. 1b). Other significant mean methylation differences (*p* < 0.01) between F0 primary tumors and F1 PDX tumors were found either in CpG island-containing probes (Fig. 1d) or probes from the intermediate HIL CpG class (Additional file 1: Figure S1e) but not in the high HIL CpG class, indicating some non-random effect on methylation of CpG-containing probes.

Based on global DNA methylation patterns, all PDX tumors were clustered together with their respective PDX type (PDX-36, -37, and -56), irrespective of their propagated generation (F1, F2, or F3) (Fig. 1e). Notably, unsupervised clustering revealed that the methylation patterns of primary tumors from patients 36 and 37 were more similar to each other than their corresponding PDX tumors as shown by the close hierarchical clustering between these two tumors (Fig. 1e). The reason for such clustering could be the fact that primary patient tumors include human stromal and endothelial cells as well.

After analyzing the number of differentially methylated CpG sites among primary tumors and PDX tumors from F1 to F3, we found only 2604 CpG sites in F1, 4349 sites in F2, and 4606 sites in F3 that were significantly differentially methylated (*p* < 0.01) in comparison with the F0 primary tumors. These results indicate that only 0.66–1.17 % of the 392,317 CpG sites were differentially methylated in primary versus PDX tumors (Fig. 1f). Moreover, a very low number of CpG sites (0.001–0.002 % of total CpG sites analyzed) was significantly differentially methylated (*p* < 0.01) among different generations of PDX tumors (F1 versus F2 or F2 versus F3 tumors) (Fig. 1f). Finally, global methylation patterns of all patient tumors and PDXs were verified by bisulfite pyrosequencing of the global methylation marker ALU-Yb8 (Additional file 1: Figure S1f), showing similar genome-wide methylation patterns between F0 and F3. In addition, the global methylation patterns of biological replicates of PDX-36 tumors from generation F3 (n = 3) were compared to each other and found to be highly correlated to each other (r = 0.94–0.96, *p* < 0.001) (Additional file 1: Figure S1g). In conclusion, these results indicate that genome-wide methylation between PDX tumors and their corresponding primary patient tumors were very similar, with only some small changes found in F1 tumors in specific CpG- enriched regions.

### Effect of treatment with demethylating agent DAC or cisplatin on the global DNA methylome of PDX tumors

**Fig. 2:**
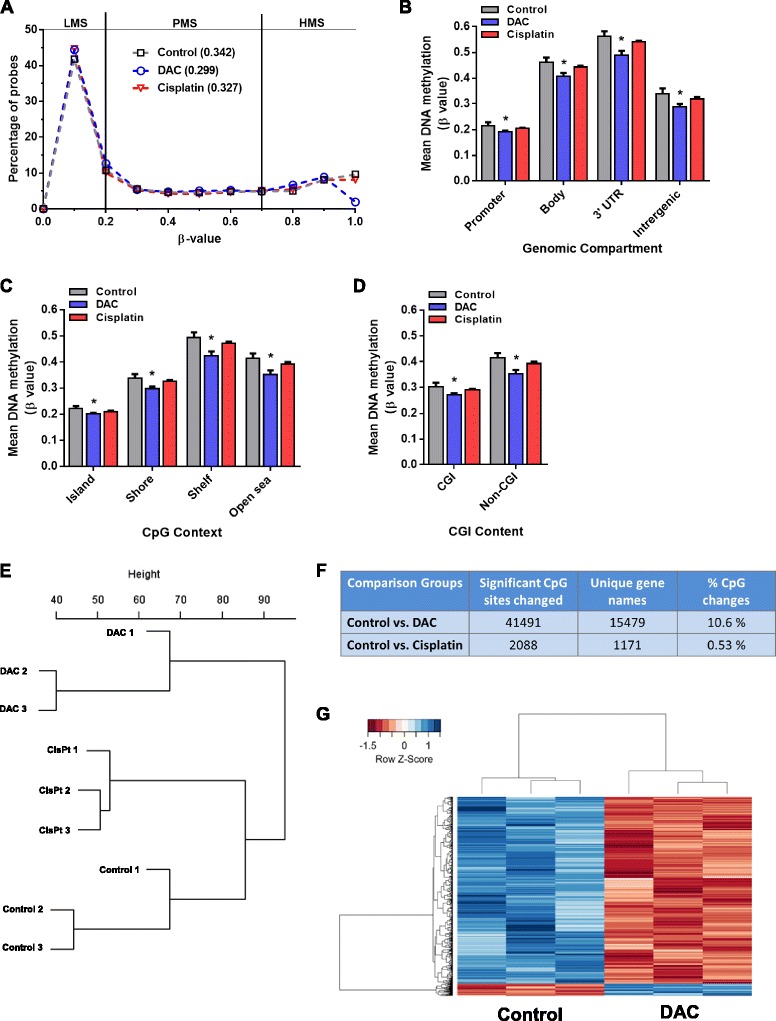
Distribution of methylated CpG sites in HGSOC PDX tumors treated with decitabine (*DAC*) and cisplatin. **a** β-values are grouped in 0.1 increments and the percentage of probes is represented for each treatment group. The mean β value for each treatment group is shown between *parentheses*. Lowly, partially and highly methylated sites are indicated as LMS, PMS, and HMS, respectively. **b**–**d** DNA methylation level of each treatment group according to the genomic compartment (**b**), CpG context (**c**), and CpG island (*CGI*) content (**d**). Each *bar* represents mean DNA methylation β value ± SD. A Student’s *t*-test was performed compared to vehicle treated PDX tumors (F0); *p < 0.01. **e** Unsupervised clustering dendrogram showing the relationship of CpG probes between all the treatment groups. **f** Significant CpG sites in comparison with different sample types and their percentage compared to total CpG sites analyzed. **g** Supervised clustering analysis of significantly changed CpG sites (*p* < 0.01) in PDX-36 treated with DAC compared to vehicle-treated controls (n = 3 mice in each group)

PDX-36 mice (n = 3) were treated with DAC and we observed a profound significant demethylation effect in genome-wide CpG probes (mean β value) of DAC-treated PDX-36 tumors (DAC = 0.299) compared to vehicle-treated tumors (control = 0.342) (Fig. 2a). Notably, DAC treatment mainly affected highly methylated probes (HMS, β > 0.7; Fig. 2a; Additional file 2: Figure S2b). Demethylation effects of DAC were observed at all regions of CpG probes, irrespective of genomic compartment, CpG context, and HIL CpG class (Fig. 2b–d; Additional file 2: Figure S2c). These results indicate that DAC treatment causes global demethylation in PDX tumors. Bisulfite pyrosequencing of global DNA methylation surrogate marker ALU Yb8 and LINE-1 confirmed our findings, revealing significant (*p* < 0.01) demethylation of DAC-treated PDX tumor DNA compared to vehicle-treated PDX tumor DNA (Additional file 2: Figure S2d).

No major demethylation effect in genome-wide CpG probes (mean β value) of cisplatin-treated PDX 36 tumors (cisplatin = 0.327) was observed compared to vehicle-treated PDX 36 tumors (control = 0.342) (Fig. 2a; Additional file 2: Figure S2b). Furthermore, there was no significant difference in mean DNA methylation between the probes of cisplatin-treated and vehicle-treated PDX tumors at any genomic location irrespective of CpG context and content (Fig. 2b–d; Additional file 2: Figure S2c). Bisulfite pyrosequencing of global DNA methylation surrogate marker LINE-1 and ALU Yb8 in PDX tumors confirmed our findings (Additional file 4: Figure S3a, b). Furthermore, no significant differences were observed for methylation of LINE-1 and ALU Yb8 in ovarian cancer cell lines when treated with either cisplatin or carboplatin compared to untreated controls (Additional file 4: Figure S3c, d). Notably, unsupervised cluster analysis of all CpG sites showed that PDX tumors clustered together dependent on the treatment used (Fig. 2e). This apparently indicates that DNA methylation patterns are similarly affected per specific therapy.

Methylation analysis at the single CpG probe level revealed approximately 41,491 CpG sites (10.6 % of total CpG sites analyzed) that were significantly differentially methylated (*p* < 0.01) in DAC-treated PDX tumors compared to control PDX tumors (Fig. 2f; Additional file 2: Figure S2e). Supervised clustering analysis of the significantly (*p* < 0.01) differentially methylated CpG sites (n = 41,491 sites) showed that the majority of sites (97.6 %) were demethylated in DAC-treated compared to vehicle-treated tumors (Fig. 2g). Interestingly, global DNA demethylation of PDX tumors is comparable to the demethylation effect of DAC as observed in tumor DNA from patients in a recent clinical trial with DAC [39] (Additional file 2: Figure S2f). In stark contrast, only 0.53 % of total analyzed CpG sites, comprising 2088 sites, were significantly differentially methylated (*p* < 0.01) in cisplatin-treated PDX tumors compared to vehicle-treated ones (Fig. 2f). Of 2088 CpG sites, 61 % of CpG sites showed hypomethylation and 39 % showed hypermethylation in cisplatin-treated tumors in comparison with vehicle-treated ones (Additional file 2: Figure S2g). In conclusion, these results show a marginal effect of cisplatin but a strong demethylation effect of DAC in clinically relevant PDX models.

### Identification of novel epigenetically regulated genes and pathways in PDX tumors

**Fig. 3:**
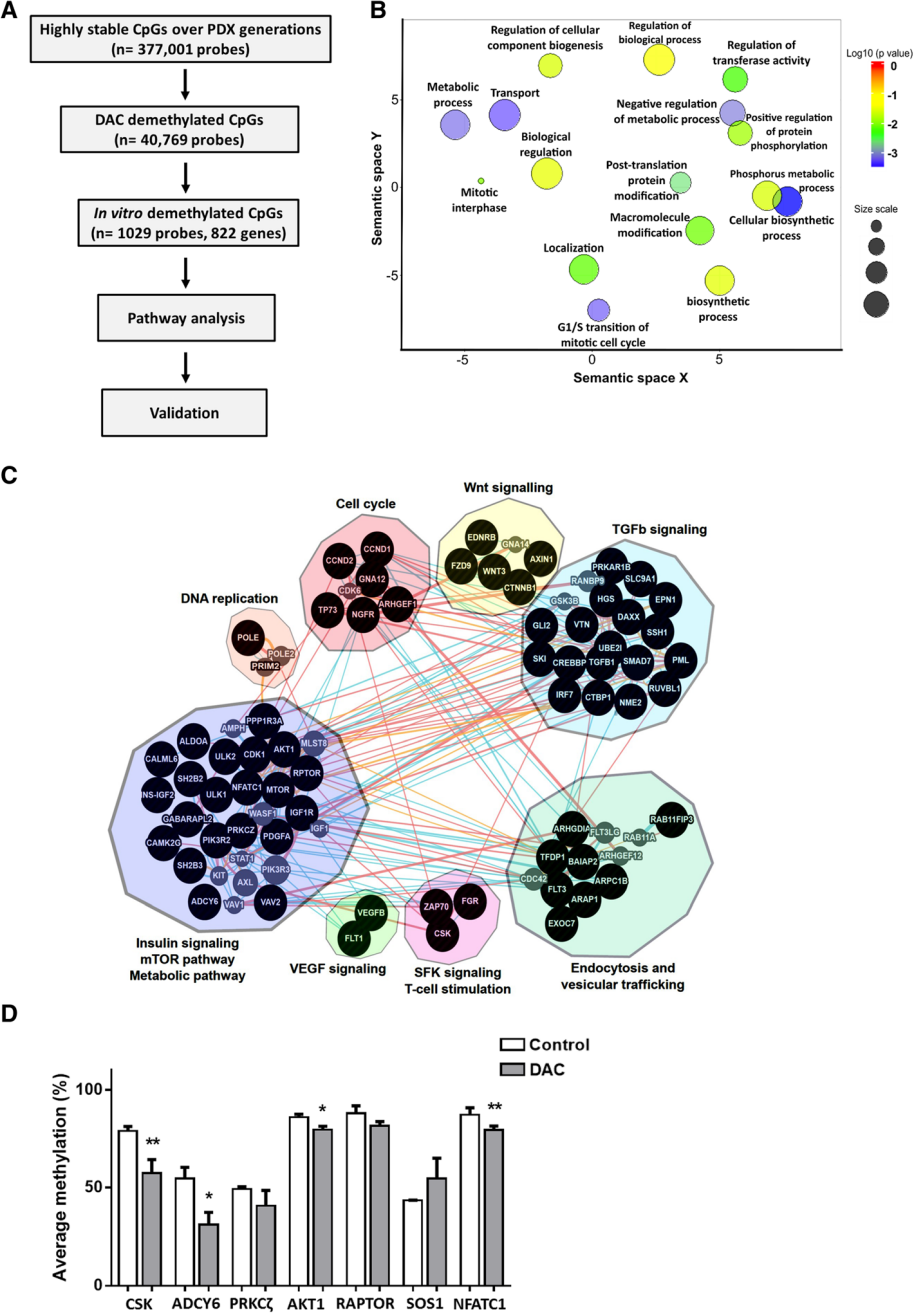
Identification of putative epigenetically regulated key genes and pathways related to ovarian cancer using PDX tumors. **a** Systematic strategy to identify CpG sites of novel putative epigenetically regulated genes. **b** Gene ontology terms enriched for biological processes using the candidate genes identified in the systematic strategy (n = 822). **c** Interactive functional association network based on predictive gene function and pathways using the same candidate genes (n = 822) by GeneMania (http://www.genemania.org/). *Blue lines* indicate related pathway connection; *orange lines* represent predicted interactions and *red lines* physical interactions. **d** Verification of seven DAC-affected genes using bisulfite pyrosequencing. Mean methylation (%) ± SD of respective genes for different analyzed CpG sites; *p < 0.05, **p < 0.01

DAC-treated PDXs showed diminished growth compared to control tumors (Additional file 5: Figure S4a), indicating that we used an effective dose of DAC. This observation allowed us to investigate changes in epigenetically regulated genes and pathways that are related to DAC-induced growth inhibition. To identify genes that are putatively epigenetically regulated, i.e., DAC-induced demethylation-sensitive genes, we selected those CpG sites that were stable at the methylome level over all generations (F1, F2, and F3) in all three PDX models (in total 377,001 CpG sites) (Fig. 3a). Of those 377,001 CpG sites, we found 40,769 were demethylated in DAC-treated PDX-36 tumors (Additional file 5: Figure S4b). This comparison resulted in 40,769 CpG sites that were stable over propagated generations and can be modulated by DAC treatment. Since we would like to validate the identified putative CpG sites functionally using ovarian cancer cell lines, we compared these PDX tumor-based 40,796 CpG sites with the DAC-sensitive CpG sites of SKOV3 cells. This resulted in 1029 CpG sites comprising 822 genes affected by DAC treatment in vivo as well as in vitro (Fig. 3a; Additional file 5: Figure S4c; Additional file 6: Table S2).

To identify the potential biological function of these 822 genes effectively demethylated by DAC treatment, we first performed GO-based functional enrichment analysis using DAVID [32]. The major biological process-related GO terms were metabolic process, cellular transport, biosynthetic process, mitotic cell cycle, cell locomotion, transferase activity, and post-translational modifications (Fig. 3b). Subsequently, pathway enrichment analysis using KEGG, Wiki pathways, and pathway common databases revealed several enriched pathways, including mTOR pathway, insulin signaling, cellular metabolic pathway, TGF-β signaling, Wnt pathway, cell cycle, Src family kinases signaling, DNA replication, and vesicular trafficking pathways (Fig. 3c; Additional file 7: Table S3). We selected seven genes from different pathways for further validation: *CSK* (Src family kinase signaling), *ADCY6* (metabolic pathway), *PRKCζ*, *AKT1*, *RAPTOR* (insulin and mTOR pathway), *SKI* (TGF-β signaling), and *NFATC1* (T-cell stimulation). Five out of these seven genes were successfully validated by bisulfite pyrosequencing comparing DNA from PDX-36 tumors treated with DAC or vehicle (Fig. 3d).

### Validation of C-terminal Src kinase (*CSK*) as a candidate gene for ovarian cancer treatment

**Fig. 4:**
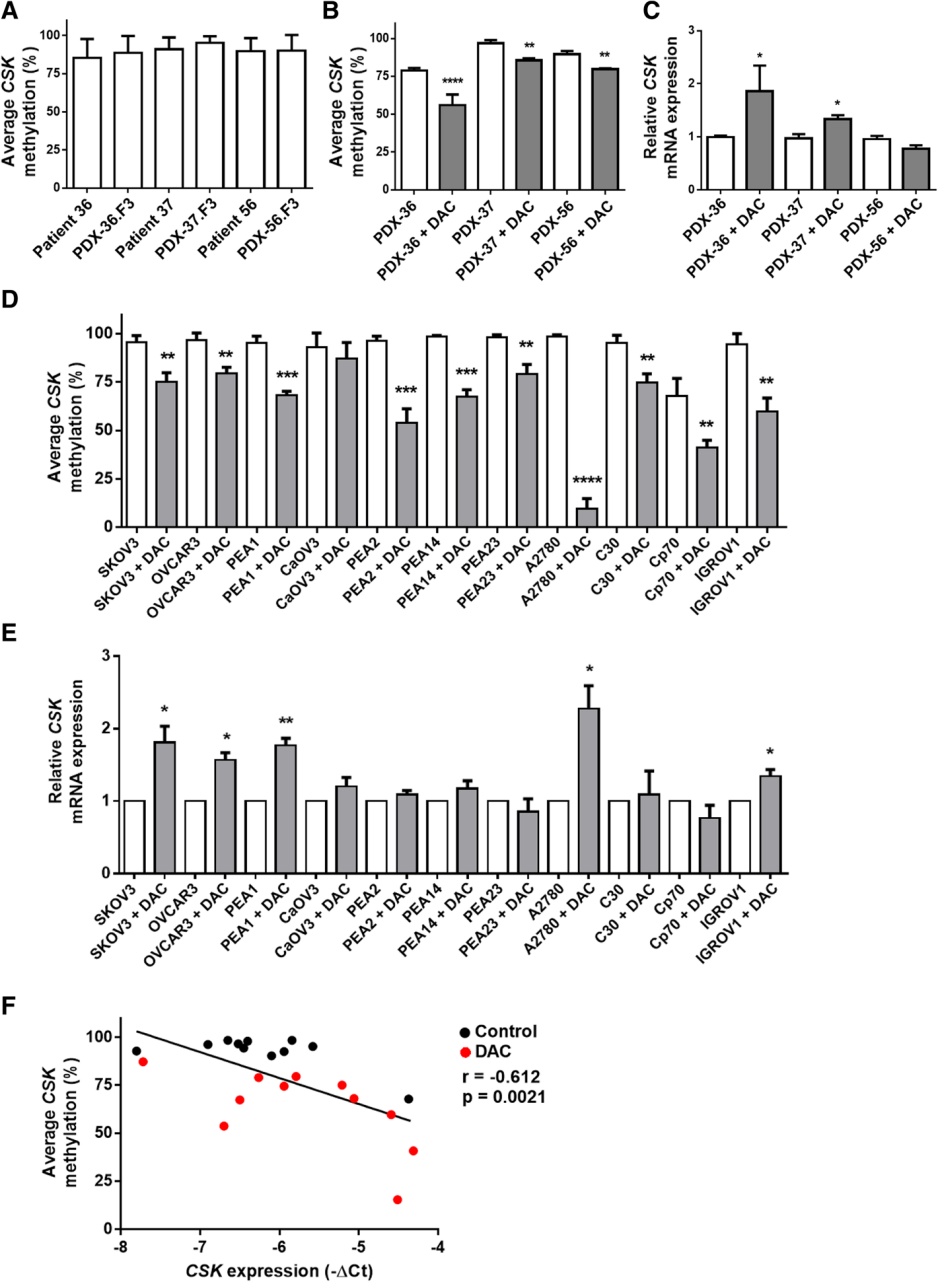
Validation of *CSK* as a novel putative epigenetically regulated gene in HGSOC. **a** Mean methylation (%) ± SD of *CSK* in patient and F3 generation tumors in three different PDXs. **b** Mean methylation (%) ± SD of *CSK* in three different F3 generation PDX tumors treated with DAC or vehicle (PDX-36, -37 and -56, n = 3 mice in each group); **p < 0.01, ****p < 0.0001. **c** *CSK* mRNA relative expression in three different F3 generation PDX tumors treated with DAC or vehicle (PDX-36, -37 and -56, n = 3 mice in each group) using qRT-PCR; *p < 0.05. **d** Bisulfite pyrosequencing of *CSK* in a panel of ovarian cancer cell lines (n = 11), untreated and treated with DAC (1 μM) for 72 h. Mean methylation (%) ± SD of *CSK* for three analyzed CpG sites; **p < 0.01, ***p < 0.001, ****p < 0.0001. **e** qRT-PCR for *CSK* mRNA expression in the same ovarian cancer cell line panel, untreated and treated with DAC (1 μM) for 72 h. Relative fold induction ± SD of *CSK* for three independent experiments; *p < 0.05, **p < 0.01. **f** Correlation analysis of methylation and expression of ovarian cancer cell lines (n = 11) treated or untreated with DAC, showing an inverse correlation between methylation and expression

Among these five successfully validated genes, we selected the C-terminal Src kinase (*CSK*) gene for further investigation, mainly because of the significantly highest demethylation effect on *CSK* after DAC treatment in PDX-36 tumors, the relevance of CSK biological function as a negative regulator of non-receptor Src family kinases, and the involvement of CSK in many key signaling pathways along with its anti-tumor activity [39, 40]. As expected, the methylation status of *CSK* among all PDX generations was stable in all different models using bisulfite pyrosequencing (Fig. 4a). Demethylation of *CSK* by DAC treatment was confirmed in all three PDX models, with the strongest effect in PDX-36 tumors (Fig. 4b). In DAC-treated PDX-36 and -37 tumors, efficient demethylation of *CSK* was accompanied by a clear induction of *CSK* gene expression (Fig. 4c).

For further validation, a large panel of ovarian cancer cell lines (n = 11) was treated with DAC for three days and the methylation status of *CSK* was analyzed using bisulfite pyrosequencing. All cell lines showed high *CSK* methylation levels (72–99 %), which decreased significantly (*p* < 0.01–0.0001) after DAC treatment (Fig. 4d). Subsequently, we found significant upregulation of *CSK* expression levels (*p* < 0.05) in SKOV3, OVCAR3, PEA1, A2780, and IGROV1 cells (Fig. 4e). Moreover, an inverse correlation (r = −0.612, *p* < 0.0021) between methylation and gene expression of *CSK* was found in the ovarian cancer cell lines (Fig. 4f). In summary, these results show that *CSK* is an epigenetically regulated gene with demethylation leading to higher gene expression, both in ovarian cancer PDX models as well as in cell lines.

**Fig. 5:**
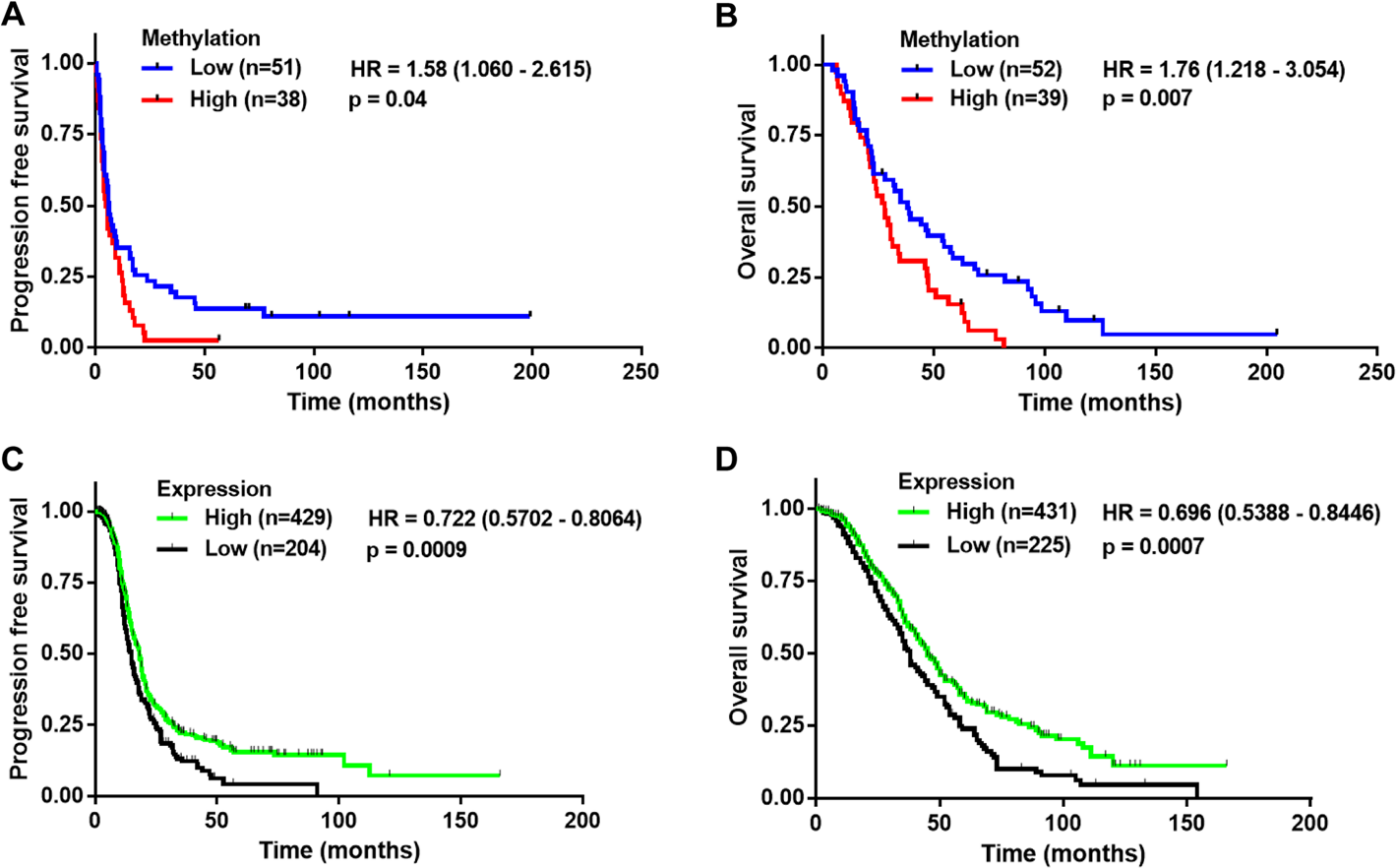
Prognostic evaluation of *CSK* methylation and expression in HGSOC patients. **a, b** Kaplan–Meier plots showing PFS (**a**) and OS (**b**) for the two patient groups defined based on *CSK* methylation using a Cox proportional hazard model in HGSOC cohorts (n = 89 and n = 91, respectively). **c, d** Kaplan–Meier plots showing PFS (**c**) and OS (**d**) for the two patient clusters based on *CSK* expression using a Cox proportional hazard model in HGSOC cohorts (n = 633 and n = 656, respectively)

Finally, to evaluate the possible clinical significance of *CSK* methylation, we used a patient database of advanced stage HGSOC patients (n = 91) who were treated with platinum-based chemotherapy and whose tumors were used to generate genome-wide methylation profiles using 450 K Infinium methylation arrays. High methylation of *CSK* (β value >0.9) was associated with a presumably poor response to platinum-containing chemotherapy of HGSOC patients as indicated by a shorter PFS (hazard ratio = 1.58 (1.060–2.615), *p* = 0.040) and with a worse OS (hazard ratio = 1.55 (1.033–2.567), *p* = 0.007) (Fig. 5a, b). The high methylation levels observed in these HGSOC patients were in agreement with the methylation levels found in the PDX tumors as well as in the ovarian cancer cell line panel. To determine the prognostic value of CSK expression in HGSOC, we used a large patient cohort of advanced stage HGSOC patients (n = 651) who were treated with platinum-based chemotherapy. High expression of CSK (probably resulting from less DNA methylation) was associated with presumably better response to platinum-containing chemotherapy of HGSOC patients as indicated by a longer PFS (hazard ratio = 0.72 (0.570-0.806), *p* = 0.0009) and with an improved OS (hazard ratio = 0.70 (0.539-0.845), *p* = 0.0007) (Fig. 5c, d). This analysis indicates the prognostic value of *CSK* methylation and expression in advanced stage HGSOC patients.

## Discussion

Our study for the first time shows that HGSOC PDX tumors are epigenetically stable comparing primary tumors with their subsequent PDX generations. Only 0.66–1.17 % of the total methylated CpG sites significantly changed in HGSOC PDX tumors during propagation. While cisplatin treatment did not alter the DNA methylation pattern, treating these PDX models with DAC significantly reduced tumor growth and was accompanied by significant changes in methylation of CpG sites. Further validation and subsequent pathway analysis revealed enrichment of several biological pathways (e.g., the Src family kinase pathway) in HGSOC that were affected by DAC treatment. Expression of *CSK*, a negative regulator of non-receptor Src family kinases, is epigenetically regulated and can be upregulated by DAC treatment in several HGSOC PDXs and cell lines. Moreover, we show that *CSK* methylation and expression have prognostic value in HGSOC patients.

There is growing evidence that HGSOC PDX models not only recapitulate the histology of patients’ tumors but also maintain the heterogeneity of them to some extent [12, 13]. However, their utility in epigenomics studies has not been assessed yet. In HGSOC, frequent aberrant epigenomic alterations, including DNA methylation, with less somatic mutations [2] present DNA methylation as a suitable target for future epigenetic cancer therapy. Finding novel and robust epigenetically regulated genes and pathways warrants suitable preclinical models with better prediction value for therapeutic targets and therapy response. Cell lines and cell line-based xenografts are known to be more homogenous models but with the lack of representative prediction of drug responses [41]. Moreover, continuous propagation of cell lines induces many epigenetic changes and HGSOC cell lines are, therefore, epigenetically far from patient tumors [42]. Until now global DNA methylome analysis has been performed on PDX models of just a few cancer types, including head and neck, small cell lung, and colon cancer and osteosarcoma [17–19]. All previously reported studies were limited by having low numbers of PDX samples and not including propagation-related, trans-generational comparisons. So far, only osteosarcoma and colon PDXs have been used to compare global trans-generational methylation patterns up to the second generation [19]. In line with our observations, the methylome of osteosarcoma and colon cancer PDXs was very similar to the primary tumor with, on average, only 2.7 % difference in the assayed CpG sites. In this study, we used only subcutaneously implanted PDX models, which do not have the advantages of orthotopic implanted models that have the same anatomic microenvironment of patients’ tumors and resemble their metastatic behavior [10]. However, the generation of orthotropic xenografts is more labor-intensive and expensive and complex surgery and imaging methods are required to monitor tumor growth [10]. Therefore, we used subcutaneous implantation methodology, achieving not only high take rates but also PDX tumors that histologically and genomically mimicked the patients’ tumors [12]. Nevertheless, it would be of interest to compare the global methylome of subcutaneously implanted and orthotropic implanted PDX models.

Current knowledge regarding the effect of epigenetic drugs like demethylating agents on DNA methylation patterns in PDX models is obscure. Our study presents the first results of DAC treatment on the methylome of HGSOC PDX tumors. We observed a global demethylation effect of DAC treatment at all CpG sites irrespective of their genomic location, with 10.6 % significantly demethylated CpG sites. We also verified these results by a decrease in DNA methylation of the global methylation markers LINE-1 and ALU Yb8. Similar global demethylation effects (e.g., LINE-1 and ALU Yb8 as well as the total percentage of demethylated CpGs) were also observed in peripheral blood mononuclear cells, ascites, and tumor DNA in platinum-resistant ovarian cancer patients treated with DAC in a phase II clinical trial [43]. Many of the significantly demethylated genes in DAC-treated PDXs in our study were related to cell–cell adhesion, MAPK, mTOR, cytokine- and chemokine-related pathways, cell–matrix adhesion, NFKB, and other related pathways. Most of these pathways were also found to be altered in DAC-treated ovarian cancer patients. However, less similarity was observed at the gene level [43, 44]. Concisely, the effects of DAC on HGSOC PDX models resemble the effects of this agent observed in patients. Hence, HGSOC PDX models can be utilized for analyzing the effects of novel epigenetic cancer therapies.

Notably, we observed DAC-induced tumor growth inhibition in all three HGSOC PDX models. Therefore, we focused on finding putative genes and/or pathways whose demethylation might be responsible for such tumor growth inhibition. Consequently, our search for novel epigenetically regulated key genes and pathways related to DAC treatment in HGSOC PDXs led to the identification of *CSK*. CSK is known for its role as a negative regulator of non-receptor tyrosine Src family kinases, including c-Src, c-Fgr, Lyn, c-Yes, and others [39]. CSK phosphorylates these kinases, leading to an inactive conformation of kinases and decreased downstream signaling [39, 45]. CSK has been found highly expressed in normal organs, while its reduced expression and concomitant increased c-Src activity were reported in many cancer types, including hepatocellular carcinoma and prostate cancer [46, 47]. We found that high expression as well as low methylation of *CSK* in advanced stage HGSOC patients was related to better PFS and OS. Moreover, Src and other Src kinases have been reported to be overexpressed in advanced stage ovarian cancer [48, 49]. Emerging data are supporting the key role of Src family kinases in many carcinogenic processes, including tumor growth and metastasis in ovarian and colon cancer [50, 51]. Hence, they are being considered as suitable targets for ovarian cancer treatment in combination with standard chemotherapy [51, 52]. It has, however, been reported that selective inhibition of Src in ovarian cancer could lead to enhanced expression of other Src family kinases and related pathways [53]. CSK overexpression actually causes inhibition of in vivo tumor growth and metastasis in colon cancer cell lines [54]. Therefore, it is tempting to speculate that reversion of epigenetically silenced *CSK* or induction of CSK expression in ovarian cancer might lead to an adequate suppression of Src family kinases and consequently less tumor growth. Thus, more in-depth functional validation of CSK is warranted to study how this protein is involved in chemoresponses and OS of ovarian cancer patients.

## Conclusions

We show that genome-wide DNA methylation in HGSOC PDX models is largely stable during propagation in mice. The methylome of PDX tumors can be efficiently affected with the demethylating agent DAC. Using this model, we have identified novel epigenetically regulated genes, such as *CSK*, and related pathways. Our results encourage the application of PDXs for further cancer epigenomics studies.

## Additional files

Additional file 1: Figure S1.
**a** Clinicopathological features of transplanted HGSOC tumors. **b** Representation of patients and their corresponding PDX tumor samples used in this study. **c** All preprocessing of 450 K data of each sample. **d** DNA methylation level of each sample type according to HIL CpG classes [38] for PDX-36, -37, and -56 for all generation tumors (F0, F1, F2, and F3), *p* < 0.01. **e** Global distribution of 450 K methylation probes of the raw data of PDX-36, -37, and -56 for all generation tumors (F0, F1, F2, and F3). **f** Validation of global methylation using bisulfite pyrosequencing of ALU-Yb8 in PDX samples. Each *bar* represents average methylation (%) ± SD of five CpG sites for ALU-Yb8 in the indicated PDX samples. **g** Correlation heat map of PDX-36 biological replicates (n = 3) of generation F3 based on their genome-wide CpGs β values. Pearson correlation coefficients are shown in each heatmap box. (PDF 620 kb)
Additional file 2: Figure S2.
**a** Systematic representation of F3 PDXs and their treatment schedule. **b** Global distribution of 450 K methylation probes of the raw data of PDX-36, untreated or treated, tumor samples. **c** DNA methylation level of each sample type according to HIL CpG classes [38] for PDX-36, untreated or treated tumor samples; **p* < 0.01. **d** Validation of global methylation using bisulfite pyrosequencing of ALU-Yb8 and LINE-1 in PDX samples. Each *bar* represents average methylation (%) ± SD of five CpG sites for ALU-Yb8 and LINE-1 in the indicated PDX samples and SKOV3 cells; ***p* < 0.001, *****p* < 0.00001. **e** Significantly changed CpG sites (*p* <0.01) in PDX-36 and altered CpG sites (∆β value > ﻿0.﻿﻿1)﻿ of SKOV3 cells after DAC treatment. **f** Comparative analysis of significantly differentially methylated CpG probes (percentages) in ovarian cancer patients [43] and PDX tumors treated with DAC in comparison with untreated control ones. **g** Supervised clustering analysis of significantly genome-wide demethylated sites (*p* < 0.01) in PDX-36 treated with cisplatin compared to vehicle-treated controls (n = 3 mice in each group). (PDF 567 kb)
Additional file 3: Table S1. All ovarian cancer cell lines and their culture conditions. (XLSX 13 kb)
Additional file 4: Figure S3.
**a, b** Validation of global methylation using bisulfite pyrosequencing of LINE-1 (**a**) and ALU-Yb8 (**b**) in PDX samples treated with either vehicle or cisplatin (4 mg/kg/week) for 4 weeks (n = 3 mice per group). Each *bar* represents average methylation (%) ± SD of five CpG sites for LINE-1 and ALU-Yb8 in the indicated PDX samples. **c, d** Effect of cisplatin and carboplatin treatment on global methylation in various ovarian cancer cell lines using bisulfite pyrosequencing LINE-1 (**c**) and ALU-Yb8 (**d**). Each *bar* represents average methylation (%) ± SD of five CpG sites for LINE-1 and ALU-Yb8 in the indicated cell lines. Each cell line was treated with either cisplatin or carboplatin at the indicated dose for 72 h. (PDF 225 kb)
Additional file 5: Figure S4.
**a** Change in tumor growth (percentages) of PDX-36, -37, and -56 during treatment with DAC (2.5 mg/kg, thrice per week) or vehicle for 4 weeks (n = 3 mice per group). **b** Comparative analysis of significant differentially methylated CpG sites among DAC-treated PDX tumors over samples from all generations. This analysis revealed 40,769 CpG sites that remained stably methylated over all generations (F0, F1, F2, and F3) and can be significantly demethylated by DAC treatment. **c** Comparative analysis of significant differentially methylated CpG sites (*p* <0.01) among DAC-treated PDX tumors over altered CpG sites (∆β value > 0.﻿3) of DAC-treated SKOV3 cells. We used stringent criteria (∆β value > 0.﻿3) for altered CpG sites for DAC-treated SKOV3 in order to select better candidate genes for further analysis. (PDF 605 kb)
Additional file 6: Table S2. Final list of the top 1029 CpG probes and their β values of DAC-treated ovarian cancer PDXs and control ones with full annotation information of probes. (XLSX 438 kb)
Additional file 7: Table S3. Table for GO enrichment and pathway analysis. (XLSX 37 kb)

## Abbreviations

BMIQ: Beta-mixture quantile
CSK: C-terminal Src kinase
DAC: Decitabine (2′-deoxy-5-azacytidine)
GO: Gene ontology
HGSOC: High-grade serous ovarian cancer
HMS: Highly methylated site
OS: Overall survival
PDX: Patient-derived xenograft
PFS: Progression-free survival
PMS: Partially methylated site
qRT-PCR: Quantitative reverse transcriptase polymerase chain reaction
SD: Standard deviation.
