# Supplementary figures and images for: Genome-wide methylation profiling of ovarian cancer patient-derived xenografts treated with the demethylating agent decitabine identifies novel epigenetically regulated genes and pathways
Source: Genome Med. 2016 Oct 20;8:107. doi: 10.1186/s13073-016-0361-5 (PMC5072346; doi:10.1186/s13073-016-0361-5)

Figure S1

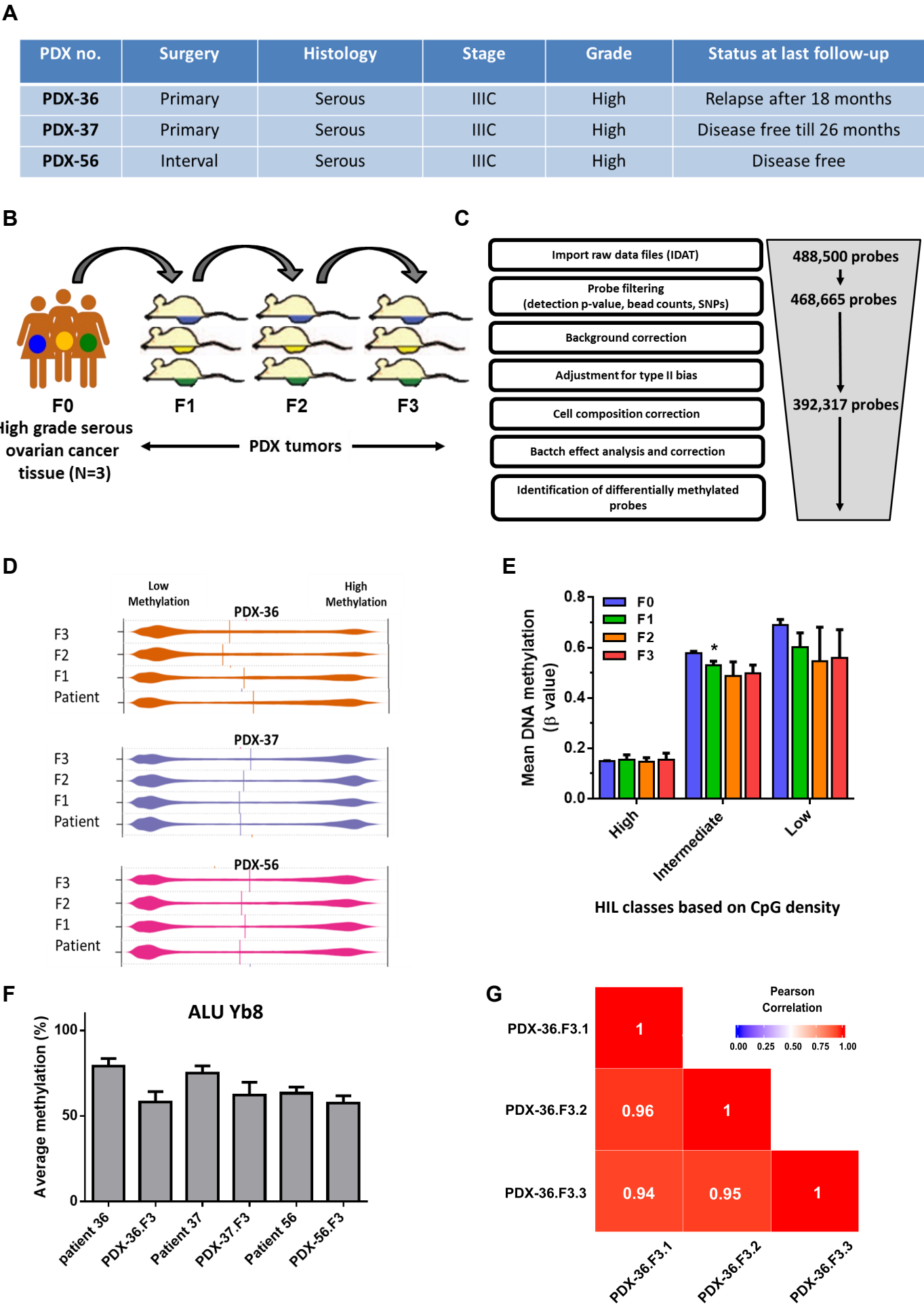

Supplement: Additional file 1: Figure S1. — a Clinicopathological features of transplanted HGSOC tumors. b Representation of patients and their corresponding PDX tumor samples used in this study. c All preprocessing of 450 K data of each sample. d DNA methylation level of each sample type according to HIL CpG classes [38] for PDX-36, -37, and -56 for all generation tumors (F0, F1, F2, and F3), p < 0.01. e Global distribution of 450 K methylation probes of the raw data of PDX-36, -37, and -56 for all generation tumors (F0, F1, F2, and F3). f Validation of global methylation using bisulfite pyrosequencing of ALU-Yb8 in PDX samples. Each bar represents average methylation (%) ± SD of five CpG sites for ALU-Yb8 in the indicated PDX samples. g Correlation heat map of PDX-36 biological replicates (n = 3) of generation F3 based on their genome-wide CpGs β values. Pearson correlation coefficients are shown in each heatmap box. (PDF 620 kb) [file 13073_2016_361_MOESM1_ESM.pdf]

Figure S2

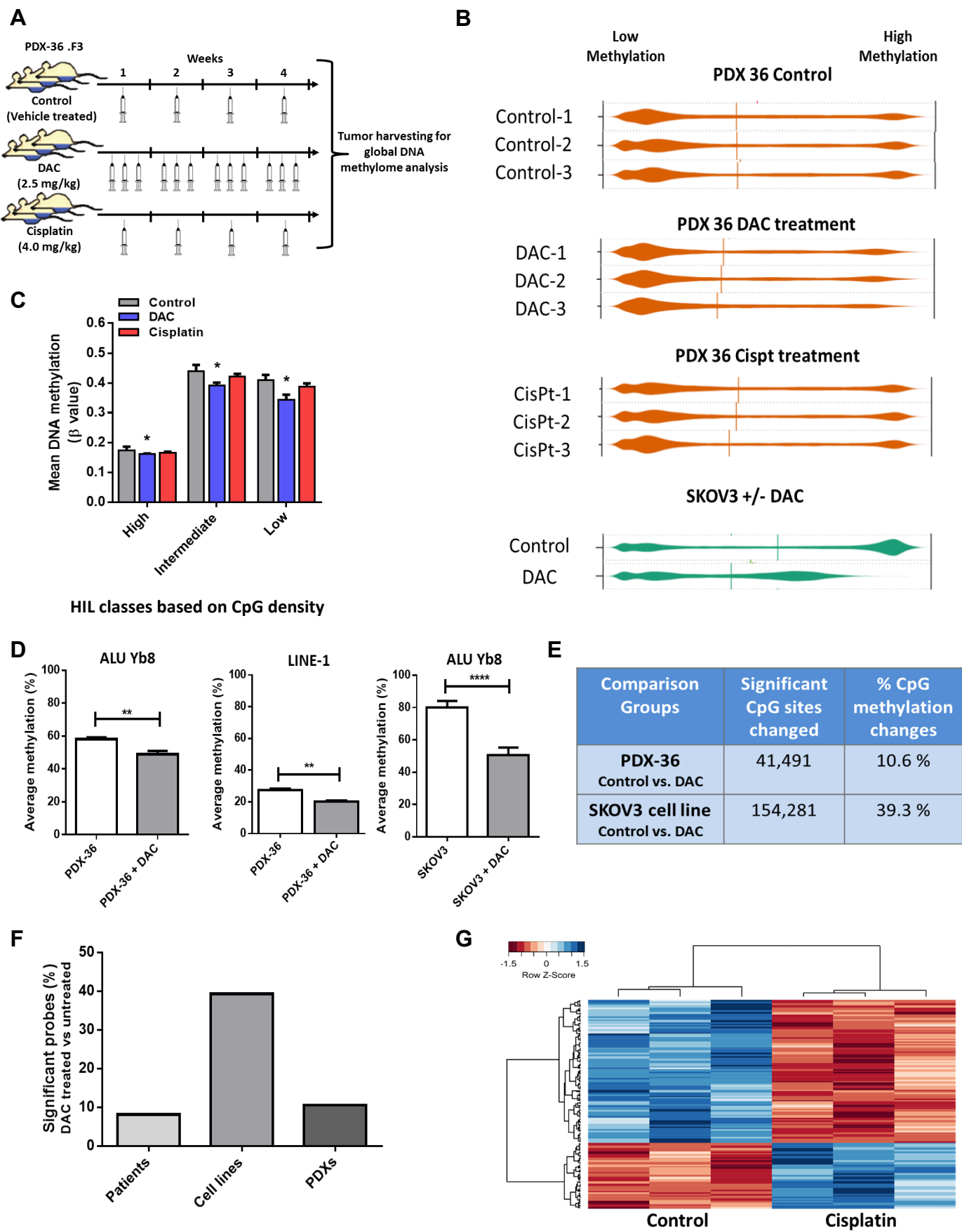

Supplement: Additional file 2: Figure S2. — a Systematic representation of F3 PDXs and their treatment schedule. b Global distribution of 450 K methylation probes of the raw data of PDX-36, untreated or treated, tumor samples. c DNA methylation level of each sample type according to HIL CpG classes [38] for PDX-36, untreated or treated tumor samples; *p < 0.01. d Validation of global methylation using bisulfite pyrosequencing of ALU-Yb8 and LINE-1 in PDX samples. Each bar represents average methylation (%) ± SD of five CpG sites for ALU-Yb8 and LINE-1 in the indicated PDX samples and SKOV3 cells; **p < 0.001, ****p < 0.00001. e Significantly changed CpG sites (p <0.01) in PDX-36 and altered CpG sites (∆β value > ﻿0.﻿﻿1)﻿ of SKOV3 cells after DAC treatment. f Comparative analysis of significantly differentially methylated CpG probes (percentages) in ovarian cancer patients [43] and PDX tumors treated with DAC in comparison with untreated control ones. g Supervised clustering analysis of significantly genome-wide demethylated sites (p < 0.01) in PDX-36 treated with cisplatin compared to vehicle-treated controls (n = 3 mice in each group). (PDF 567 kb) [file 13073_2016_361_MOESM2_ESM.pdf]

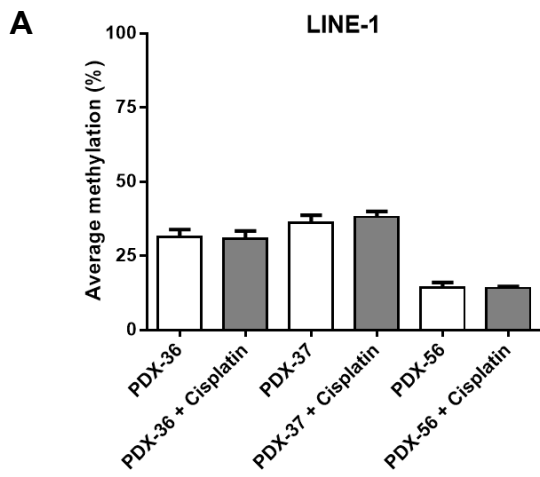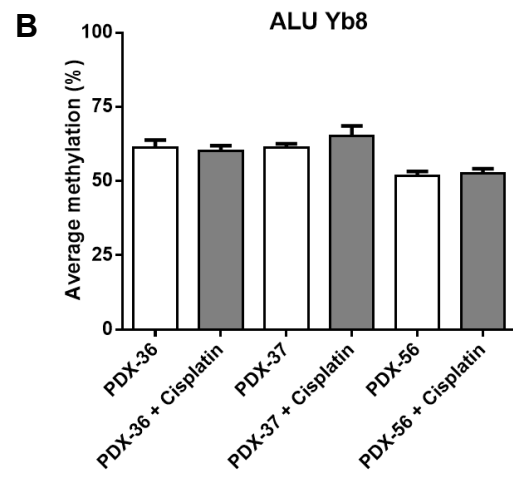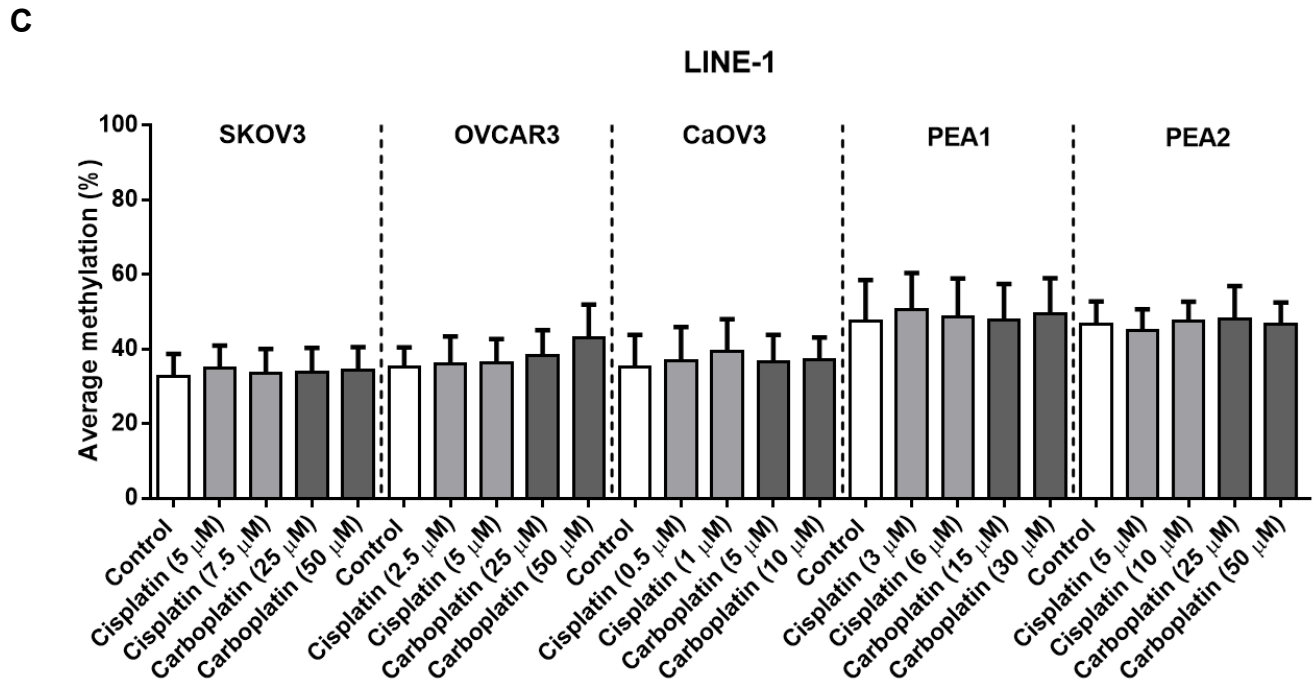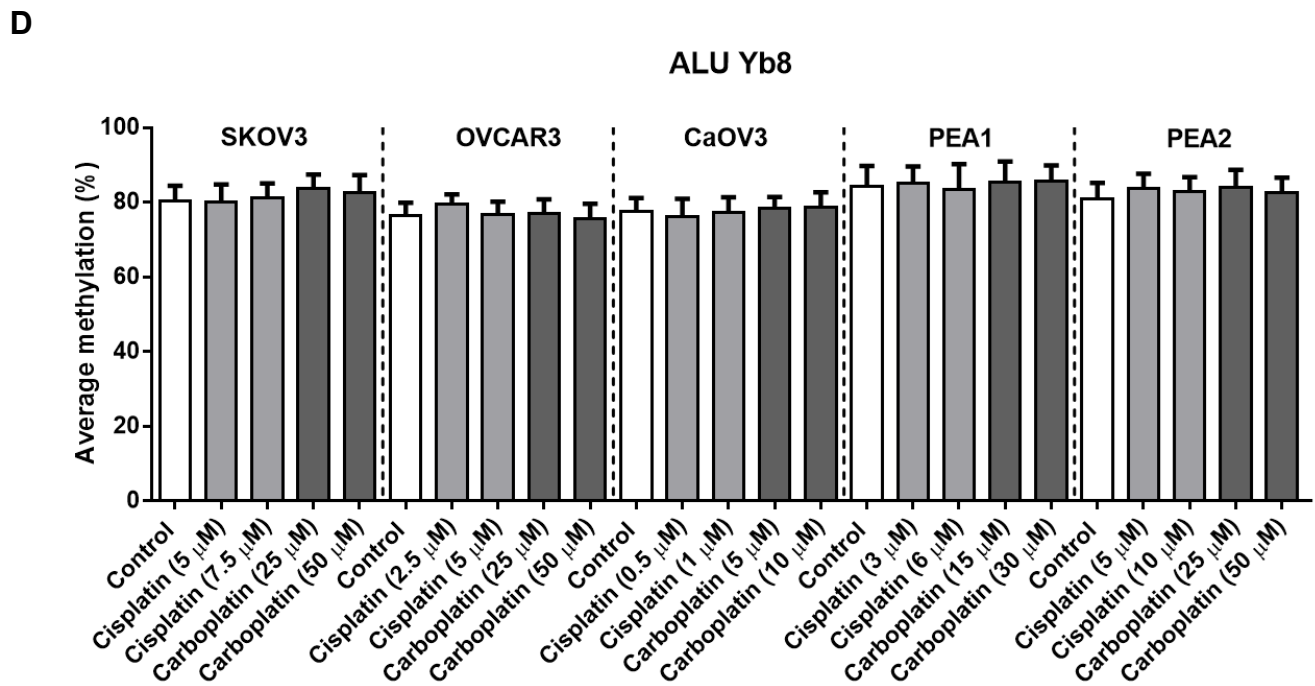

Supplement: Additional file 4: Figure S3. — a, b Validation of global methylation using bisulfite pyrosequencing of LINE-1 (a) and ALU-Yb8 (b) in PDX samples treated with either vehicle or cisplatin (4 mg/kg/week) for 4 weeks (n = 3 mice per group). Each bar represents average methylation (%) ± SD of five CpG sites for LINE-1 and ALU-Yb8 in the indicated PDX samples. c, d Effect of cisplatin and carboplatin treatment on global methylation in various ovarian cancer cell lines using bisulfite pyrosequencing LINE-1 (c) and ALU-Yb8 (d). Each bar represents average methylation (%) ± SD of five CpG sites for LINE-1 and ALU-Yb8 in the indicated cell lines. Each cell line was treated with either cisplatin or carboplatin at the indicated dose for 72 h. (PDF 225 kb) [file 13073_2016_361_MOESM4_ESM.pdf]

Figure S4

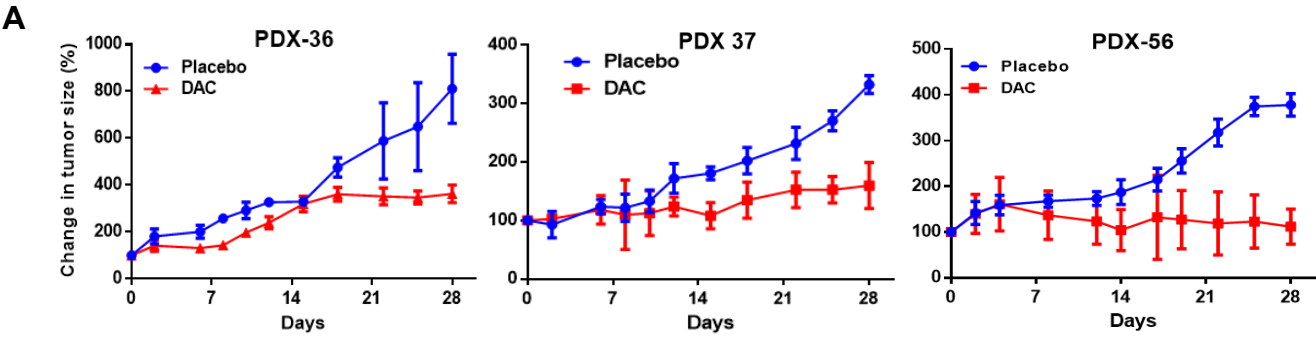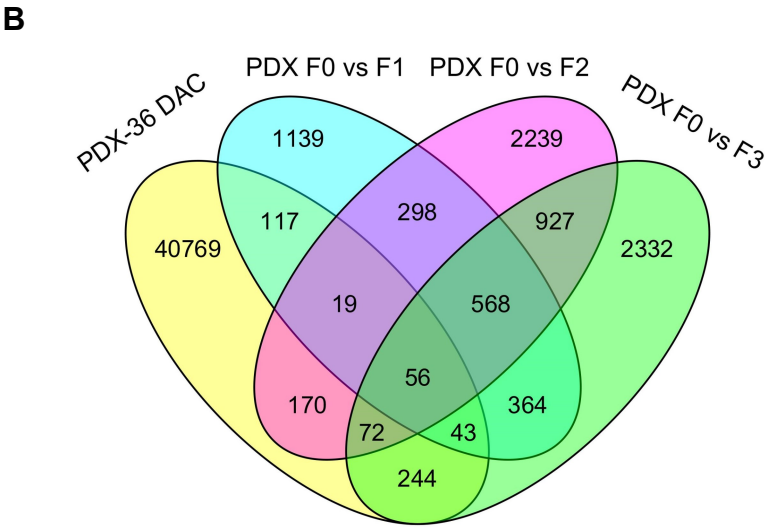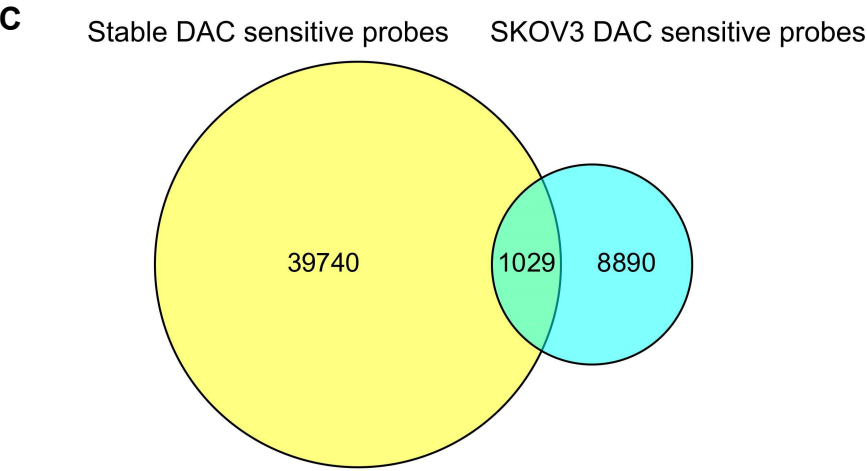

Supplement: Additional file 5: Figure S4. — a Change in tumor growth (percentages) of PDX-36, -37, and -56 during treatment with DAC (2.5 mg/kg, thrice per week) or vehicle for 4 weeks (n = 3 mice per group). b Comparative analysis of significant differentially methylated CpG sites among DAC-treated PDX tumors over samples from all generations. This analysis revealed 40,769 CpG sites that remained stably methylated over all generations (F0, F1, F2, and F3) and can be significantly demethylated by DAC treatment. c Comparative analysis of significant differentially methylated CpG sites (p <0.01) among DAC-treated PDX tumors over altered CpG sites (∆β value > 0.﻿3) of DAC-treated SKOV3 cells. We used stringent criteria (∆β value > 0.﻿3) for altered CpG sites for DAC-treated SKOV3 in order to select better candidate genes for further analysis. (PDF 605 kb) [file 13073_2016_361_MOESM5_ESM.pdf]
